# The Influence of Celebrity Endorsement on Food Consumption Behavior

**DOI:** 10.3390/foods10092224

**Published:** 2021-09-19

**Authors:** Cristina Calvo-Porral, Sergio Rivaroli, Javier Orosa-González

**Affiliations:** 1 Departamento de Empresa, Facultad Economia y Empresa, Universidade da Coruña, 15071 A Coruña, Spain; jorosa@udc.es; 2 Dipartimento di Scienze e Tecnologie Agro-Alimentari, Alma Mater Studiorum-Universitá di Bologna, 40126 Bologna, Italy; sergio.rivaroli@unibo.it

**Keywords:** food, celebrity, endorsement, consumer behavior, purchase intention, premium price

## Abstract

“Is consumer food behavior influenced by celebrity endorsement?”. This question remains unsolved despite celebrities constantly recommending different products in their social media networks. Much of the literature on celebrity endorsement focuses on the characteristics of celebrities influencing consumers’ behavior, but there is scarce research about how celebrity endorsements about food and food products influence consumers’ behavior. In this context and based on the source credibility and source attractiveness models, as well as on the match-up theory, this study aims to examine whether consumers’ food purchase intention and consumers’ willingness to pay a premium price is influenced by celebrity endorsement. For this purpose, an empirical study is developed through Structural Equation Modeling (PLS-SEM) based on data gathered from 316 consumers who read celebrity recommendations. Findings report that consumers are most influenced in their food consumption behavior by the congruence between the celebrity endorsement and the product being recommended, and by the celebrity credibility. Interestingly, celebrity recommendations show a similar influencing pattern both for consumers’ food purchase intention and consumers’ willingness to pay a premium price for food. The major contribution of this research is to show that congruence is the main route by which celebrity endorsement influences food consumption behavior.

## 1. Introduction

Celebrity endorsement is one of the most popular tools in marketing communication and has been profusely used in order to promote brands, products and services. In fact, the use of celebrity endorsement as a method of communication has increased significantly in recent years and has become a relevant phenomenon worldwide. The reason is its great effectiveness as a communication tool, its positive impact on consumer attitudes and behavioral intentions for the endorsed good [1], as well as its influence on consumers’ purchase behavior [2], brand awareness and brand recognition [3]. Further, celebrity endorsement is a communication tool that makes products and brands more attractive and appealing to potential customers; accordingly, many consumers are willing to purchase and pay a premium price for the products their favorite celebrities endorse. Similarly, nowadays celebrities are gaining influence among consumers, given the increase in their presence in social media, and evidence shows that recommendations by celebrities on social networking platforms are increasingly important to change consumers’ behavior.

In this context, research on celebrity endorsement has traditionally focused on the source characteristics of the endorser and on the transfer of meanings between the endorser and the recommended product or brand. In fact, important efforts have been made in marketing research to understand the underlying factors that drive celebrity endorsement influence on consumers’ behavior. Previous research on celebrity endorsement is mostly developed from three theoretical approaches—source credibility [4] and source attractiveness models [5], affect transfer theory [6], and match-up theory [7]—which aim to examine the bases of the influence of celebrity endorsement on consumers’ behavior.

However, more research is required to examine what factors of celebrity endorsement influence food consumption behavior and whether celebrity endorsement influences consumers’ willingness to pay a premium price for the endorsed food. Therefore, to the best of the authors’ knowledge, this research is one of the first to empirically investigate the influence of celebrity food endorsement on consumers’ behavior, and more precisely, the attributes of celebrities that are influencing consumers’ food purchase intention and their willingness to pay a premium price. More precisely, the purpose of the present study is to empirically investigate the influence of celebrity characteristics, namely credibility, trustworthiness, expertise, attractiveness and congruence with the endorsed product on consumers’ food behavior.

## 2. Literature Review

### 2.1. Celebrity Endorsement as a Communication Tool

Celebrity endorsement enjoys great popularity as a marketing communication tool. In recent decades, companies and marketers have used celebrities to promote their products, services and brands; today, with the increasing development of the internet and social media, celebrity endorsement is becoming an increasingly important medium for communicating with consumers.

According to [8], a celebrity endorser can be defined as “any individual who enjoys public recognition and who uses this recognition on behalf of a consumer product, by appearing with it in an advertisement”. Therefore, celebrity endorsement refers to a marketing communication tool that represents a person who uses his/her public recognition and popularity in order to promote the consumption, use or sales of a product, service or brand [8]. Later, authors such as [9] conceptualized the term celebrity as “any person who enjoys high social reputation and public recognition including movie stars, artists, singers, athletes, politicians, and so on”. So, it can be stated that celebrities are individuals who are successful in their professions, who enjoy public recognition and who have media attention.

Prior research shows that for most consumers, celebrities represent idealized role models, and many individuals enjoy learning, reading and keeping updated about celebrities’ lives [10]. Consequently, celebrities are effective endorsers, due to the connotations for their aspirational reference groups [6]; further, previous researchers indicate that celebrity endorsement is an effective way to enhance purchase intentions and purchase behavior [2]. 

Interestingly, the receptivity of consumers to the messages delivered by celebrities is particularly high for product categories with high social and psychological risks [1,11] and for product categories involving factors of discriminating taste, other individual’s opinions and self-image [12]. In addition, there is abundant research indicating that the characteristics of the celebrity endorser influence the endorsement effectiveness.

### 2.2. Celebrity Endorsement of Food Products

Celebrity endorsement changes consumer behavior and attitudes, but these influences may change depending on the product category, and this effect is known as the significance of the type of product endorsed. In other words, in addition to endorser-specific factors, the product category and product type being endorsed constitute a determining factor of the celebrity’s impact on consumers’ behavior [11].

More precisely, low-involvement products and fast-moving consumer goods purchased through routine decision-making and chosen quickly by consumers without a deep information search or comparison are effectively endorsed through likable spokespersons [13].

Regarding the celebrity endorsement of food products, it should be noted that previous research mostly examined and found empirical support for the effectiveness of celebrity endorsement in food choice and food intake [14,15]. Most previous studies focused on marketing campaigns using celebrity endorsement that encouraged healthy dietary habits such as the consumption of vegetables, fruit or the consumption of water [16,17]. Further, food and beverage companies particularly use celebrity endorsement of athletes in order to promote food products’ consumption specially targeting children and young consumers [18]. Finally, recent research shows that the appearance of a celebrity endorsing food products posted on online social media such as Instagram has a greater impact on consumer behavioral responses than any other type of endorser [19].

## 3. Research Hypotheses Development

**Figure 1:**
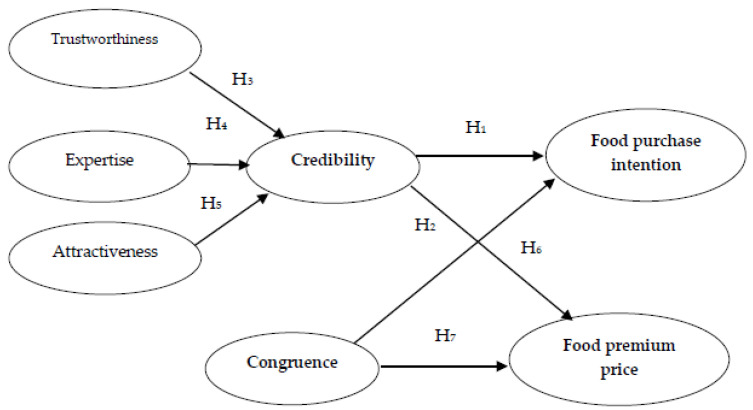
Conceptual model for food purchase intention and willingness to pay a premium price influenced by celebrity endorsement.

This study develops a conceptual model to examine the influence of celebrity characteristics on consumers’ food purchase intention and their willingness to pay premium prices for food items recommended by celebrities (Figure 1). The conceptual model is theoretically grounded on the source credibility [4] and source attractiveness models [5], as well as on match-up theory [7]. In addition, following [20], we have considered that credibility could be operationalized as a second-order construct of trustworthiness, expertise and attractiveness, as depicted in Figure 1.

### 3.1. Credibility of Celebrities

The term credibility can be understood as the extent to which a source is perceived as possessing significant knowledge or enough experience to offer an unbiased judgment [21]; therefore, credibility outlines whether an individual recognizes a claim as being true, unbiased and honest. Accordingly, endorser credibility could be defined as “the extent to which a source is perceived as possessing expertise relevant to the communication topic; and in turn, can be trusted to give an objective opinion on the subject” [22].

The effectiveness of celebrities in influencing consumers’ behavior was first explained through the source-credibility model. This theoretical model, developed by [4] and further substantiated by [22], proposes that a source should encompass two requirements in order to be credible, namely trustworthiness and expertise. Therefore, in this theoretical framework, credibility could be considered as a multidimensional construct composed of the dimensions expertise and trustworthiness.

According to the source-credibility model, the persuasiveness of a message greatly depends on the credibility of the message sender; and in turn, the power of the celebrity endorsement is based on the perceived level of trustworthiness and expertise associated with the specific endorser [1]. So, the message will have a more positive impact if the source—or the endorser—of a message is perceived as “credible”. Likewise, the source-credibility model was extended in order to include the attribute of the attractiveness of the source [22]; so that the effectiveness of the endorsement will be influenced by the attractiveness of the endorser.

The impact of endorser credibility on consumer attitude change and consumer behavior has received great attention in the marketing area. In general terms, credibility has proven to have a substantial positive influence on consumers’ attitudes and intentions [1,2], further influencing attitude towards products and brands and consumer purchase intention [3,23]. Similarly, those celebrity endorsements that are perceived as being more authentic or natural, such as an image of an endorser using a specific product in his/her daily routine on social media, have higher persuasive effects [24]. In addition, prior research reports that source credibility has a positive effect on willingness to pay a premium price [3,25]. Therefore, the following research hypotheses are presented:

**Hypotheses** **1.** *The credibility of the celebrity positively influences consumers’ purchase intention*.

**Hypotheses** **2.** *The credibility of the celebrity positively influences consumers’ intention to pay a premium price*.

### 3.2. Trustworthiness of Celebrities

Trustworthiness addresses the question of whether an individual could be considered believable [26]; accordingly, the concept of trustworthiness refers to the extent to which consumers believe the endorser has integrity and is honest [1,22]. Similarly, and regarding the trustworthiness of celebrities, this concept can then be defined as the perceived “willingness of the celebrity to make valid assertions” [8].

According to the source credibility model [4], trustworthiness is an attribute underlying source credibility that influences consumers’ attitudinal change [22]. Likewise, a celebrity endorser who is perceived as trustworthy is more likely to influence consumers’ attitudes and behavioral intentions [22] and will have a higher persuasive influence on consumers compared to other social influences [27]. Therefore, the celebrity’s trustworthiness is a key factor, since consumers get more easily influenced by an individual whom they trust [12].

In addition, the positive influence of the celebrity’s trustworthiness on consumer attitudes and purchase intention has been reported by [28] or by [29]. Hence, considering all the stated above, the following hypothesis is posed:

**Hypotheses** **3.** *The trustworthiness of the celebrity positively influences the celebrity credibility*.

### 3.3. The Expertise of Celebrities

Expertise refers to the message sender’s knowledge, experience and skills; and accordingly, expertness could be defined as “a persuasion cue that triggers individuals to use cognitive heuristics, such as statements made by experts that can be trusted” [30]. In addition, expertise describes the individual’s level of knowledge and could be conceptualized in terms of the level of experience, knowledge and problem-solving skills that a person has in a specific area. So, an expert is able to perform at a high level in a specific domain, and becoming an expert requires practice, experience and long-term training [31].

In the context of the source-credibility model, the source expertise could be defined as the degree of an endorser’s knowledge, experience and skills in a specific area [22], or as “the skills, knowledge or experience possessed by an endorser” [1]. Similarly, according to authors such as [29], the endorser expertise refers to the extent of perceived knowledge, understanding and relevant skills of an endorser. So, the endorser’s expertise stems from his/her ability to provide information based on his/her experience, aptitude or training.

According to the source-credibility model, an endorser who demonstrates expertise is more persuasive than an endorser who does not [1,22]; in turn, the expertise of an endorser is an important factor to increase the persuasiveness of marketing communication, since the endorser expertise relates to the validity of the product claims [32]. In addition, the expertise and competence of the endorser were also found to be positively associated with consumers’ attitude and behavior [9].

Celebrities regularly share their daily routine, activities, opinions or recommendations based on previous expertise on social media networks. Accordingly, celebrities are considered to be experts in their respective fields; and individuals are more likely to take recommendations conveyed by celebrities who are perceived as experts [6]. More specifically, the expertise of a celebrity provides consumers with detailed and specific information on products, leading to a more favorable attitude towards the endorsed product [33], to attitudinal change and to higher purchase intentions [1,22,28,32].

Finally, the expertise of an endorser has been shown to have a positive influence on credibility [4] and on consumers’ purchase intention [28]. Hence, the following hypothesis is presented:

**Hypotheses** **4.** *The expertise of the celebrity with food products positively influences the celebrity’s credibility*.

### 3.4. The Attractiveness of Celebrities

The source credibility model was extended to include the attractiveness of the source [3,22]. Accordingly, the source attractiveness model proposes that the effectiveness of an endorsement is influenced by the perceived level of the physical attractiveness of the endorser [22]. The underlying motive of the influence of the endorser attractiveness is that individuals draw satisfaction from believing that they have a similar attractiveness to the endorser, and in turn, they conform to the behavior advocated by the endorser [2].

More precisely, the source-attractiveness model proposes that the effectiveness of a message depends on the source familiarity, similarity and likeability [22]: the source familiarity refers to the knowledge of the source through exposure; the source likeability could be understood as the affection for the source derived by her/his physical appearance, physical attractiveness and behavior; and finally, the source similarity refers to the perceived resemblance between the endorser and the consumer. Consequently, endorsers who are well known, liked and perceived to be similar to the consumers will be attractive; and in turn, persuasive [22]. Similarly, other authors such as [8] reported that the endorser attractiveness refers to the endorser’s appealing nature, including attributes such as physical beauty or physical attractiveness, personality and familiarity. In this vein, other authors indicate that the endorser’s attractiveness also includes an attractive lifestyle and intellectual skills [1,27].

In addition, previous research reports that attractiveness—as a dimension of the source credibility—influences the receiver of communication [5] and that physically attractive individuals facilitate attitudinal change in a more effective way [30]. More precisely, researchers of source attractiveness found that physically attractive celebrities influence consumers’ behavior and consumer attitudes favorably compared to less-attractive celebrities [11,27,34] and that the celebrity endorsers’ physical attractiveness has a positive impact on consumers’ purchase intentions [29,34]. Finally, the attractiveness of an endorser has a positive influence on credibility [22]. Hence, this hypothesis is proposed:

**Hypotheses** **5.** *The attractiveness of the celebrity positively influences the celebrity’s credibility*.

### 3.5. The Congruence between the Celebrity and the Food Product Recommended

Traditionally, the effectiveness of endorsers was thought to be developed by the attributes of the celebrity, such as the trustworthiness, expertise and attractiveness of the endorser [4,22]. However, the effectiveness of endorsement in creating favorable attitudes and intentions may be influenced by other determining factors such as the endorser and product “match-up” [7] or the congruence between the endorser and the endorsed product, that is, the fit or the degree of consistency or similarity between the endorsed product category and the endorser.

In this context, the match-up theory developed by [7] aims to provide a model in order to explain how endorser effectiveness varies according to the different product categories, suggesting that an endorser would be more effective with a high perceived congruence between the endorser and the endorsed product or brand [7]. Therefore, it can be stated that in endorsement effectiveness there is also an effect of the product type: a specific endorser could be extremely suitable for certain products and not as suitable for other product categories. So, the better the endorser–product fit, as perceived by consumers, the higher effectiveness of the endorsement [1,8,11].

Accordingly, the match-up theory [7] provides a conceptual model for celebrity endorsements and proposes that when an adequate match-up between the celebrity and the endorsed product takes place, the obtained “match-up” becomes central to the message [35], making the celebrity recommendation effective [2,11,34], and increasing the favorable impact of the celebrity endorsement [36]. More specifically, prior research highlights the importance of the perceived congruence between the celebrity’s image and the product image to influence consumers’ purchase intention [37] to enhance the positive assessment of the endorser and the product [7,34] and to produce more favorable responses toward the endorsement [36]. Therefore, the selection of an endorser based on image congruence seems to be an adequate strategy for product categories that are not particularly related to attractiveness or performance, such as food products [38].

Finally, and considering that previous studies have demonstrated the influence of a good endorser–product fit on consumers’ purchase intention [37], the following hypotheses are presented:

**Hypotheses** **6.** *The congruence between the celebrity and the endorsed food product positively influences consumers’ purchase intention*.

**Hypotheses** **7.** *The congruence between the celebrity and the endorsed food product positively influences consumers’ intention to pay a premium price*.

## 4. Methodology

### 4.1. Measurement

The measurement variables for this research were selected based on previous research on the topic. In the first place, the credibility of celebrities was measured by three items adapted from [22] and two items adapted from [39]. In the second place, to measure trustworthiness, expertise and the attractiveness of celebrities [22] scale was used; and in addition, two items were adopted from [40] to measure the celebrities’ trustworthiness. Third, the congruence between the celebrity endorser and the food product being recommended was measured using 5 items adapted from [41] and from [42]. Then, the consumers’ purchase intention and their willingness to pay a premium price were measured using a 4-item scale and a 2-item scale adapted from [26], respectively, as shown in Table 1.

### 4.2. Sampling and Fieldwork

The survey was conducted among consumers residing in Spain in June 2021. In order to prepare the research questionnaire, a pretest was carried out to determine a celebrity endorser of food products. More precisely, the celebrity endorser was selected based on several criteria. In the first place, the selected celebrity should enjoy great popularity and have a good public image among consumers residing in Spain. In the second place, the celebrity should be a local individual from Spain, given that native celebrities can generate more positive attitudinal responses [43]. In the third place, the celebrity should have a relationship with food. Therefore, following these criteria, the famous Spanish chefs Jordi Cruz and Dabiz Muñoz were chosen as the celebrities recommending food products on their social media. It should be noted that these two chefs have become part of the celebrity world, often appearing in TV shows and cookery books.

In the present study, we assume that celebrity endorsement of food products could be understood as the endorsement or recommendation of food products or food product categories—and, more precisely, unbranded food products—by celebrities. Further, in our study, the food endorsement was included as web content promoted in celebrities’ social media. That is, we examine celebrity-generated content whereby food products appear in content produced and shared by celebrities in their social media.

The obtained data were collected through random sampling using an online structured self-administered questionnaire. More precisely, the questionnaire consisted of three sections. Because the purpose of our study was to gather information about the influence of celebrity endorsement, a “yes/no” pre-screening question was incorporated in the questionnaire. This question was included in order to ensure that all the research participants read celebrity recommendations on social media. Therefore, participants who do not read celebrity endorsements were screened out of the survey.

Stimulus materials were presented to participants simultaneously with the questionnaire, showing the selected celebrities recommending different food products (Appendix A). Then, in the first section of the questionnaire, an introduction was included to explain the main purpose of the study. Next, the second section of the questionnaire incorporated questions regarding the variables under research, measured through a seven-point Likert scale with 1 indicating “strongly disagree” and 7 indicating “strongly agree”. The third part included socio-demographic questions and questions regarding food consumption. Finally, a total amount of 359 questionnaires were obtained, gathering 316 valid questionnaires; and in turn, yielding a sampling error of 5.62% at a confidence level of 95%.

The sample profile is shown in Table 2. Regarding the participants’ gender, the 49.8% of the respondents were female, whereas 50.2% were male. Similarly, the great majority of the participants (41.0%) were between 31 and 40 years old, followed by individuals with ages between 41 to 50 years old (25.9%). Likewise, in terms of education level, 48.7% of participants have university studies, while 37.8% of participants have secondary education. In terms of household average income level, 38.4% of the sample indicated a monthly income level between 1800 and 2700 EUR/month. Finally, regarding the participants’ frequency of reading celebrity endorsements, the majority of the respondents (30.8%) reported a frequency of “once a week”, followed by participants who indicate that read endorsements “several times a week” (22.6%).

### 4.3. Data Analysis

Partial Least Square (PLS) path modeling was developed for the estimation and analysis of the research hypotheses using the Smart PLS 3.0. software (SmartPLS GmbH, Ahornstr. 54, 25474 Boenningstedt, Germany) in order to examine the proposed conceptual model and to test the research hypotheses [44], and more precisely, the consistent PLS algorithm was developed. The analysis through path modeling is developed to measure the influence of the different constructs and all the possible causal relationships among them. The present research used PLS-SEM as the method for data analysis because is a causal-predictive approach to SEM (Structural Equation Modelling) that allows the analysis of various relationships simultaneously and enables the estimation of complex models and structural paths without distributional assumptions of the data [45].

## 5. Results

### 5.1. Analysis of the Measurement Model

Before developing the path analysis, the measurement model was tested for reliability, validity and internal consistency (Table 3).

In the first place, the Cronbach’s alpha values of each construct exceeded the suggested cut-off value of 0.70 [45], and constructs achieved composite reliability (CR) values higher than the commonly accepted threshold of 0.70, indicating an adequate internal consistency. In the second place, the convergent validity was analyzed through factor loadings and through the average variance extracted (AVE). On one hand, factor loadings reached values greater than the commonly accepted threshold of 0.70 [46], ranging from 0.752 to 0.895. On the other hand, the average variance extracted (AVE) values were higher than 0.50, indicating convergent validity.

Finally, the discriminant validity of the measurement scale was examined through the analysis of all the paired combinations of constructs. Following the criteria proposed by [46], the square root values of average variance extracted are higher than construct correlations (Table 4), indicating the discriminating validity of the constructs.

### 5.2. Analysis of the Structural Model

The analysis of the structural model and the relationships between constructs was developed through the coefficients of determination R^2^ (explained variance) and the effect size (f^2^) [45]. First, the criterion in order to evaluate the structural model was the coefficient of determination (R-square) of the endogenous variables; accordingly, these coefficients were measured. Our results show an R^2^ = 0.832 for food purchase intention and R^2^ = 0.757 for the willingness to pay a premium price (Table 5). This means that more than 80% of the purchase intention and more than 70% are explained by the independent variables of our conceptual models. Secondly, the effect size (f^2^) measures the strength of each variable in explaining endogenous variables. The results indicate that all the effect size values of the constructs achieve values higher than the commonly accepted threshold of 0.02 [45]. Third, the collinearity test for variance inflation factor (VIF) values achieved values lower than the accepted threshold of 5, indicating the adequacy of the structural model.

Finally, the SRMR is a measure of the structural model fit, and our results indicate that SRMR was 0.075, which does not surpass the threshold of 0.08, revealing reveals the good fit of the model. Similarly, the Chi-Square = 789.356 and NFI = 0.874 show an adequate structural model fit.

### 5.3. Analysis of the Relationships among Variables

The path coefficients, the corresponding t-values and levels of significance are shown in Table 6 for food purchase intention and for willingness to pay a premium price.

Regarding the consumers’ purchase intention, results indicate that consumers’ intention to purchase food products is positively influenced by the credibility of the celebrity and the congruence or match-up between the celebrity and food. More precisely, the congruence of the celebrity with food products was found to exert the highest influence of purchase intention (β_6_ = 0.539 **; *p* = 0.000), followed by the credibility of the celebrity endorser (β_1_ = 0.116 **; *p* = 0.002), which has a slight impact on the purchase intention.

Likewise, a positive influence was found for the celebrity trustworthiness on the credibility of the celebrity (β_3_ = 0.542 **; *p* = 0.000). Conversely, and contrary to our initial expectations, the expertise of the celebrity (β_4_ = 0.046 ^ns^; *p* = 1.388) and the celebrity attractiveness (β_5_ = 0.016 ^ns^; *p* = 0.991) do not show a significant influence on the credibility of the endorser. One possible reason for the lack of influence of the celebrity attractiveness is that—as reported in previous literature—the attractiveness of the celebrity endorser mostly influences consumer behavior in beauty-related products or in attractiveness-related products [1,36], which is not the case for food products.

Similarly, one potential explanation for the lack of significant influence of celebrity expertise may be that expertise could not be perceived as relevant by consumers when evaluating the credibility of a food endorser. One possible reason is that food products are frequently purchased and consumed by all consumers, and in turn, most of the consumers may have broad experience in food purchases. Another possible explanation is that consumers may rely more on the recommendations and perceptions of other consumers rather than on the recommendations of food experts. Further, this result is coherent with previous research indicating that the use of expert celebrities is not deemed necessary when recommending low-risk and non-financial products [12], which is the case with food. Additionally, our results are in line with [47], who reported that a trustworthy communicator is more persuasive than a communication who is not, regardless of whether he/she is an expert. Therefore, our findings suggest that as long as consumers perceive food endorsers as trustworthy, the celebrity would be perceived as credible and thus influence consumers’ purchase intention.

On the other hand, regarding the consumers’ willingness to pay a premium price for the endorsed food products, the congruence between the celebrity and food was found to have the highest impact (β_7_ = 0.496 **; *p* = 0.003); while the credibility of the celebrity shows a slightly lower impact on premium pricing (β_2_ = 0.412 **; *p* = 0.002) (Table 5). That is, the celebrity match-up with food is the more relevant variable in consumers’ willingness to pay a premium price for the endorsed food, followed by the credibility of the endorser. So, our findings suggest that consumers, when deciding whether to pay a price premium for a food product, are similarly influenced by the congruence and the credibility of the celebrity endorser. In addition, and contrary to our initial expectations, our findings do not support a significant influence of the celebrity’s attractiveness (β_5_ = 0.016 ^ns^; *p* = 0.991) on the perceived credibility by consumers, as hypothesized. According to our results, it seems that consumers do not consider the celebrity’s physical attractiveness, nor his/her expertise with food when assessing the reliability of the endorser.

Therefore, the celebrity congruence with food, followed by the celebrity credibility, is the stepwise order of the influence of the attributes of celebrities in food consumption. So, it can be stated that the better the celebrity match-up with food, and the greater credibility, the greater influence of consumers’ food consumption behavior.

Finally, our results provide support for five out of seven of the proposed research hypotheses for food consumption behavior, since Hypotheses 1, 2, 3, 6 and 7 are supported.

## 6. Discussion

This research aimed to examine what characteristics of celebrities influence food consumption behavior. The research question is the following: “Is consumer food behavior influenced by celebrity endorsement?”. According to our results the answer is, “Yes, and congruence seems to be the main route to influence food consumption behavior by celebrity endorsement”, since the celebrity congruence with the endorsed product was demonstrated to be the major factor influencing consumer behavior, followed by credibility.

More precisely, our findings show that consumers are more likely to purchase and pay premium prices for food products because of the credibility, congruence and trustworthiness of the celebrity endorsers. Therefore, these results indicate that celebrity endorsers who are considered to be congruent, honest and reliable, have integrity and are concerned about consumers are more likely to induce consumers’ food behavior. This finding is coherent with [38], who reported that when consumers perceive the image of a celebrity and a product to be congruent, the consumers are more likely to accept the endorsement message. Consequently, it can be stated that the characteristics of celebrity endorsers and their congruence with the recommended food product positively influence consumers’ food behavior, implying that celebrity endorsers serve as a relevant external information source. Therefore, one major contribution of the present research is to show that congruence is the most influential factor on consumer behavior it comes to recommending food products.

Another remarkable finding of the present research is that the attractiveness of celebrity endorsers shows no influence on credibility, or in turn on consumers’ behavior. One potential explanation for this result is that according to the match-up hypothesis [7], physically attractive endorsers have positive effects on consumers’ attitudes and consumers’ behavior only for attractiveness-related products such as personal care products, and food products do not fall into this product category. In other words, an attractive endorser will serve as an effective source of information for an attractiveness-related product or brand, while for attractiveness-unrelated products—such as food and food products—the match-up between the endorser attractiveness and the product does not take place. Similarly, and contrary to the initial expectations, the expertise of the celebrity endorser with food or cooking does not seem to influence the celebrity credibility, therefore not influencing food consumption behavior.

Another major contribution of this study, is that it provides empirical foundation of the similar pattern of the influence of celebrity recommendations on consumers’ purchase intention and their willingness to pay a premium price, since the credibility, congruence and trustworthiness attributes of a celebrity influence both outcomes.

## 7. Conclusions

This research contributes to the existing knowledge on celebrity endorsement by examining its influence on consumers’ behavior when recommending food products. More specifically, the present study reports that consumers are influenced in their food consumption behavior by celebrity endorsement through the celebrities’ congruence with the recommended product, their credibility and their trustworthiness. Therefore, celebrity endorsement could be considered an effective communication tool in the food marketing area, as long as the food endorsers are perceived as being congruent and credible by food consumers.

The findings of this research provide valuable managerial insights for food companies and food marketers. First, our findings suggest that the characteristics of celebrity endorsers have an impact on food consumption behavior, showing that congruent and credible celebrities have a positive impact both on food purchase intention and on the consumers’ willingness to pay a premium price. Interestingly, even though the source attractiveness model proposes the positive effect of celebrity attractiveness on consumers’ behavior, our findings report that attractiveness is not the best criterion in order to select celebrities for recommending food products, and it seems that it is better to use a congruent celebrity. So, in light of the results of our study, food marketers should care more and pay greater attention to celebrities’ congruence and give less importance to attractiveness and expertise of endorsers. Secondly, this research indicates that celebrity endorsement is an effective communication marketing tool for influencing food purchase intention and provides useful insights for selecting an effective food endorser, with his/her congruence with food being the main influencing factor. Finally, food marketers should keep in mind that carefully choosing a celebrity to influence food consumption behavior could contribute to increase consumers’ purchase intentions and help intensify the demand for specific food product categories. Therefore, our study provides valuable information on how celebrities contribute to consumer food purchase intention and to the proneness to pay a premium price.

This research has limitations that should be considered in future research. In the first place, both the celebrities presented as stimuli in the research are prestigious chefs, and celebrities from other areas such as television presenters or actors might have produced different results. In the second place, only male celebrities were presented in the stimuli of the study, and the use of female celebrities might have resulted in a different impact on food consumption behavior. Third, future research on the topic could examine the influence of other relevant food-related variables that are not included in our study, such as the consumer involvement with food, since different levels of food involvement might yield different results. Fourth, it would be interesting to investigate the relationship between celebrity credibility and congruence, since this link was not examined in the present research. In addition, another research limitation is that other items and measures of celebrity expertise could have influenced the results obtained, maybe reporting a significant relationship between expertise and celebrity credibility. Finally, this research investigated consumers’ response to social media recommendations, and future studies may examine the influence of food celebrity endorsement through traditional media.

## Figures and Tables

**Table 1:** Variables and indicators.

Variables	Indicators
Credibility Ohanian (1990); Martins et al. (2017)	CRED1: Celebrities recommending food products seem to be sincere CRED2: Celebrities recommending food products seem to be honest CRED3: Celebrities recommending food products seem to be reliable CRED4: Celebrity food influencers are a good reference for purchasing and consuming food products CRED5: Celebrity food influencers are credible and convincing
Trust Ohanian (1990); Terres et al. (2015)	TRUST1: I have confidence in the information/recommendations provided by celebrities regarding food products TRUST2: Celebrities show high level of commitment to the consumers TRUST3: Celebrities have high integrity when recommending food products TRUST4: Celebrities care and are concerned about consumers TRUST5: Food celebrity influencers consume the same food products they recommend
Expertise Ohanian (1990)	EXP1: Celebrities recommending food products are experienced in this area EXP2: Celebrities recommending food products are qualified in this area EXP3: Celebrities recommending food products are skilled in cooking
Attractiveness Ohanian (1990)	ATR1: The celebrity recommending the food product is attractive to me ATR2: I pay more attention towards advertisements/recommendations presented by attractive/beautiful celebrities
Congruence Speed and Thompson (2000); Dwivedi et al. (2016)	CONG1: There is a logical connection between the food product and the celebrity CONG2: There is a match-up between the food product and the celebrity CONG3: The combination of the food product and the celebrity is adequate CONG4: The images between celebrities recommending food products and the products recommended are connected CONG5: The image of celebrities recommending food products and the products recommended are closely related
Purchase intention Wiedmann et al. (2014)	PINT: I am likely to purchase food products recommended by celebrities PINT2: My willingness to purchase food products recommended by celebrities is high PINT3: I would purchase food products recommended by celebrities PINT4: Celebrity recommendations inspire me to purchase the recommended food product
Premium price Wiedmann et al. (2014)	PREM1: I would be willing to pay a premium price for food products recommended by celebrities PREM2: Food products recommended by celebrities are worth a higher price than other food products

**Table 2:** Sample description.

Variable	Category	Frequency	Percentage (%)
Gender	Male	160	50.2
	Female	156	49.8
Age	18–30	15	4.9
	31–40	130	41.0
	41–50	82	25.9
	51–60	68	21.6
	More than 60	21	6.6
Household average income (Eur/month)	900–1800	65	20.7
	1800–2700	121	38.4
	2700–4500	95	29.7
	More than 4500	35	11.2
Education level	Primary education	33	10.3
	Secondary education	119	37.8
	University studies	154	48.7
	Doctorate	10	3.2
Frequency reading celebrity recommendations	Daily	37	11.8
	Several times a week	71	22.6
	Once a week	97	30.8
	Several times a month	66	21.0
	Once a month	24	7.6
	Occasionally/Several times a year	21	6.2

**Table 3:** Factor loadings and indicators of internal consistency and reliability.

Construct	Items	Cronbach Alpha	Standardized Loadings	CR	AVE
Credibility	Cred1	0.882	0.863	0.914	0.679
	Cred2		0.804		
	Cred3		0.785		
	Cred4		0.850		
	Cred5		0.816		
Trust	Trust1	0.875	0.842	0.909	0.667
	Trust2		0.782		
	Trust3		0.832		
	Trust4		0.830		
	Trust5		0.795		
Expertise	Exp1	0.769	0.878	0.867	0.686
	Exp2		0.775		
	Exp3		0.827		
Attractiveness	Attr1	0.783	0.775	0.823	0.701
	Attr2		0.895		
Congruence	Cong1	0.857	0.832	0.898	0.637
	Cong2		0.824		
	Cong3		0.752		
	Cong4		0.818		
	Cong5		0.761		
Purchase intention	Pint1	0.867	0.843	0.910	0.716
	Pint2		0.827		
	Pint3		0.818		
	Pint4		0.894		
Premium price	Prem1	0.745	0.893	0.887	0.797
	Prem2		0.892		

**Table 4:** Correlations and discriminant validity values.

	Cred	Trust	Exp	Attr	Cong	Pint	Prem
Credibility	**0.824**						
Trust	0.503	**0.817**					
Expertise	0.531	0.662	**0.828**				
Attractiveness	0.578	0.512	0.671	**0.837**			
Congruence	0.683	0.432	0.697	0.567	**0.798**		
Purchase intention	0.661	0.612	0.615	0.512	0.602	**0.846**	
Premium price	0.639	0.658	0.684	0.652	0.550	0.616	**0.893**

Note: the correlations between constructs are shown in the off-diagonal and bold numbers in the diagonal correspond to the square roots of AVE for each construct.

**Table 5:** Structural model evaluation.

Constructs	VIF Collinearity Assessment	Confidence Intervals	Level of R^2^	f^2^ Effect Size
Credibility	4.547	0.038–0.529	0.828	0.114
Trust	4.703	0.432–0.917		0.534
Expertise	4.127	−0.072–0.435		0.056
Attractiveness	3.117	−0.110–0.275		0.046
Congruence	4.447	0.416–0.882		0.539
Purchase intention			0.832	
Premium price			0.757	

**Table 6:** Model resolution through PLS consistent algorithm.

Path Analysis	Standardized β Coefficients	t-Statistic	*p*-Value	Hypotheses Test
Credibility → Food Purchase intention	ß_1_ = 0.116 **	2.333	0.002	Hypotheses 1: Supported
Credibility → Premium price	ß_2_ = 0.412 **	2.300	0.002	Hypotheses 2: Supported
Trust→ Credibility	Β_3_ = 0.542 **	5.096	0.000	Hypotheses 3: Supported
Expertise→ Credibility	ß_4_ = 0.046 **	1.388	0.166	Hypotheses 4: Not Supported
Attractiveness → Credibility	ß_5_ = 0.016 ^ns^	0.991	0.332	Hypotheses 5: Not Supported
Congruence → Food Purchase intention	ß_6_ = 0.539 **	5.247	0.000	Hypotheses 6: Supported
Congruence → Premium price	ß_7_ = 0.496 **	2.966	0.003	Hypotheses 7: Supported
ns = no significant; ** significant (*p* < 0.05)	R^2^ (Purchase intention) = 0.832 R^2^ (Willingness to pay premium price) = 0.757			

**Figure A1:**
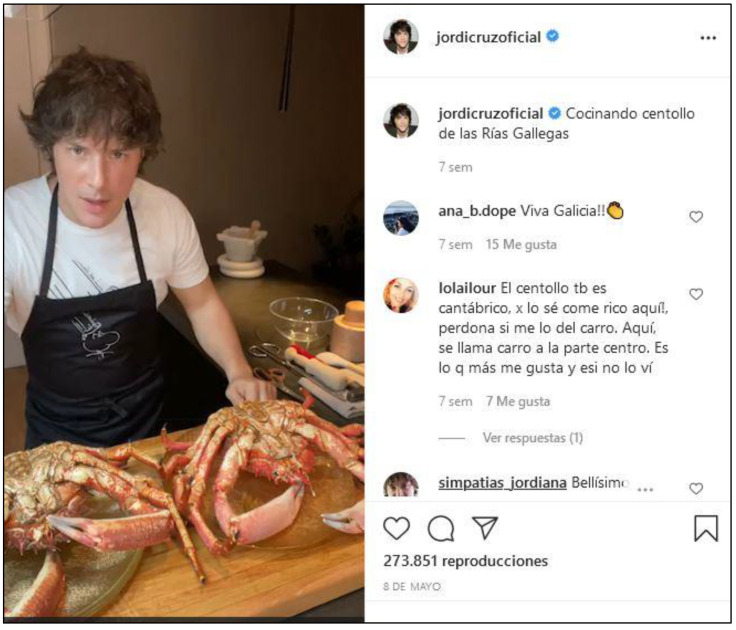
Image of the Instagram of the chef Jordi Cruz.

**Figure A2:**
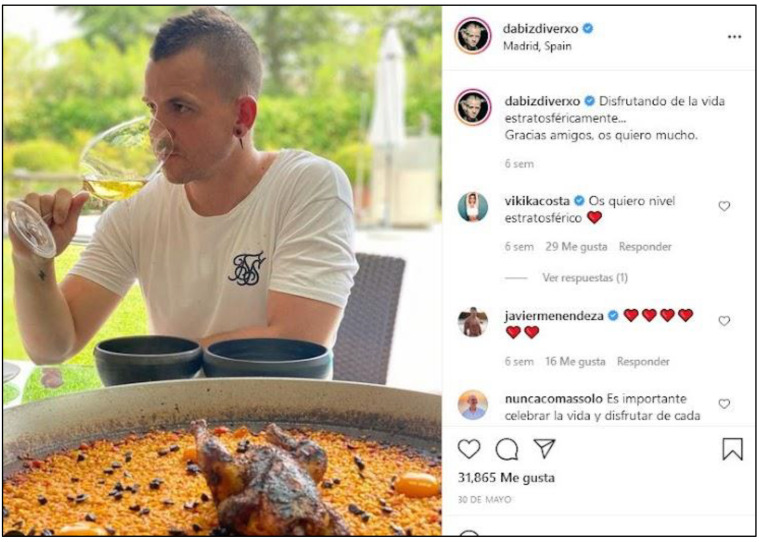
Image of the Instagram of the chef Dabiz Muñoz.

## Data Availability

Data of this research are available on request due to privacy restrictions. The data presented in this research will be available on request from the corresponding author.

## Appendix A

Stimulus materials were presented to research participants when the questionnaire was administered. The stimuli present two popular Spanish celebrity chefs recommending food products in their social media networks.
